# Structural Imbalance Promotes Behavior Analogous to Aesthetic Preference in Domestic Chicks

**DOI:** 10.1371/journal.pone.0043029

**Published:** 2012-08-14

**Authors:** Mark A. Elliott, Orsola Rosa Salva, Paul Mulcahy, Lucia Regolin

**Affiliations:** 1 School of Psychology, National University of Ireland Galway, Galway, Republic of Ireland; 2 Center for Applied Perceptual Research, Department of Human Sciences, Kyushu University, Fukuoka, Japan; 3 Center for Mind/Brain Sciences, University of Trento, Italy; 4 Department of General Psychology, University of Padua, Italy; University of Queensland, Australia

## Abstract

**Background:**

Visual images may be judged ‘aesthetic’ when their positioning appears imbalanced. An apparent imbalance may signify an as yet incomplete action or event requiring more detailed processing. As such it may refer to phylogenetically ancient stimulus-response mechanisms such as those mediating attentional deployment.

**Methodology/Principal Findings:**

We studied preferences for structural balance or imbalance in week-old domestic chicks (*Gallus gallus*), using a conditioning procedure to reinforce pecking at either “aligned” (balanced) or “misaligned” (imbalanced) training stimuli. A testing phase with novel balanced and imbalanced stimuli established whether chicks would retain their conditioned behavior or revert to chance responding. Whereas those trained on aligned stimuli were equally likely to choose aligned or misaligned stimuli, chicks trained on misaligned stimuli maintained the trained preference.

**Conclusions/Significance:**

Our results are consistent with the idea that the coding of structural imbalance is primary and even overrides classical conditioning. Generalized to the humans, these results suggest aesthetic judgments based upon structural imbalance may be based on evolutionarily ancient mechanisms, which are shared by different vertebrate species.

## Introduction

**Figure 1:**
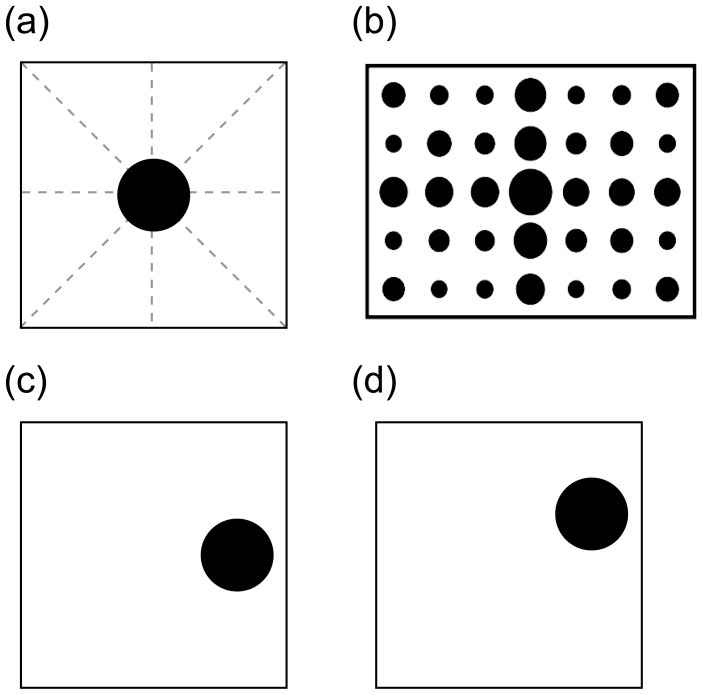
Compositional dynamics: disks and frame. In (a) a black disk is centrally aligned central to a square frame and at the intersection of all lines defining an invisible structural skeleton of directed perceptual force (defined by the square frame – the structural skeleton is illustrated here by dashed light gray lines). In the center of the frame the disk appears balanced and at rest relative to the frame. After (2), (b) shows locations within a rectangular frame judged by observers to afford greater to lesser stability (denoted respectively by larger to smaller circles). As can be clearly seen the intersection of the central vertical and horizontal meridians affords the greatest stability, while stability is afforded by locations along lines of force similar to those illustrated in (a). (c) and (d) illustrate disks located proximal to the frame. In both the disk lies proximal to the right frame edge as if suspended. However (c) appears more stable than (d), which in terms used by (1) appears restless. Whilst (c) lies on one horizontal line of force, (d) is misaligned relative to neighboring horizontal and diagonal lines.

Visual compositions exhibiting a structural misalignment of one or more components relative to other aspects of the composition may receive greater interest than simpler compositional structures because of the structural tension afforded by misalignment. Structural misalignment affords a sense of imbalance, which can, in turn, be sufficient to afford a sense of motion in which the misaligned item strives towards a location in the scene at which it appears balanced. Rudolf Arnheim [1] illustrated this with reference to framed items, deliberately positioned aligned or misaligned relative to an invisible structural skeleton of directed perceptual forces defined by their frame (illustrated in Figs. 1a and 1b). In Fig. 1c a black disc is positioned on a line of force and appears stable or “at rest” because its position is in harmony with the structure of the frame. However, in Fig. 1d the disk is misaligned relative to the structural skeleton and appears imbalanced, pulling towards the center of the frame.

Structural misalignment may afford tensions that are motion or action related because the location of the misaligned item suggests its action, activity or behavior is as yet incomplete. This characteristic could encourage prioritized or enhanced processing as, for example, is illustrated by the Zeigarnik effect [2]. This describes how people remember incomplete or interrupted tasks better than completed tasks. However in the case of compositional misalignment, prioritization is more likely to result from the deployment of attentional rather than mnemonic mechanisms with the aim of prioritizing for the purpose of enhanced perceptual processing. This means that structural misalignment encourages attentional engagement in order to facilitate a more efficient perceptual resolution of the inherent structural imbalances in the scene towards a resolved or completed composition or action [3]. Enhanced attentional and perceptual processing are very similar to, and may be sophisticated examples of the phylogenetically ancient stimulus-response mechanisms that mediate the basic orienting reflex and learning in organisms such as *Paramecium*, *Amoeba* and *Aplysia* [4]–[7]. If structural misalignment encourages a response mediated by evolutionary ancient mechanisms, evidence of a preference to respond to misalignment may present itself in the behavior of other species. This leads to the possibility that the preference judgments made by humans may not be entirely unique and instead, may share critical similarities with those made by animals. Traits that are widespread across the animal kingdom are likely to present an adaptive value and to increase the fitness of individuals bearing the trait. The adaptive value of a response bias toward structural misalignment is likely to be based in the necessity to prioritize objects with kinetic energy and as such be able to accurately predict their behaviour, intention or eventual resting state or position. For example, objects could be animate creatures seen in the moments preceding the beginning of a voluntary movement, whose “imbalanced posture” reveals the imminence of an action. They could also be physical objects located in an unstable position and as such likely to move, for instance to fall or tumble over. Preferential attention toward movement is nearly omnipresent in the animal kingdom, and the animal model we are employing in the present study (the young domestic chicken) has already proved to be sensitive to particular forms of movement indicative of the presence of biologically relevant objects [8], [9].

To examine this we investigated the influence of structural alignment/misalignment on choice behavior in domestic chicks *(Gallus gallus domesticus*). Chicks demonstrate visual preferences that closely resemble those made by humans [10]–[12] while perceptual phenomena, including visual illusions, also appear to be perceived in the same way as by humans [13]. Sophisticated social cognition that includes the ability to recognize and adopt behavior relevant to conspecifics is evident [14], [15] as well as an abstracted extension of the same faculty, with chicks demonstrating innate preference for particular stimulus configurations such as faces [16]. While newly-hatched chicks peck preferentially to symmetrical stimuli after some visual experience, visually naive chicks demonstrate a spontaneous preference for asymmetric patterns [10]. This offers a more specific indication of an innate primacy to respond to structural misalignment that may be related to focal-attentional deployment and might indeed be related to the resolution of ambiguities in the display. If this were so, it might be argued, that chicks orient to and attempt to resolve whatever seems to them to be imbalanced in a scene leading to the prediction that chicks would show an above average interest to misaligned items similar to those illustrated in Fig. 1. We tested this hypothesis, expecting that training on a stimulus misaligned relative to a major axis of symmetry will produce a more consistent tendency to approach and peck a similarly misaligned configuration during generalization to novel stimuli.

**Figure 2:**
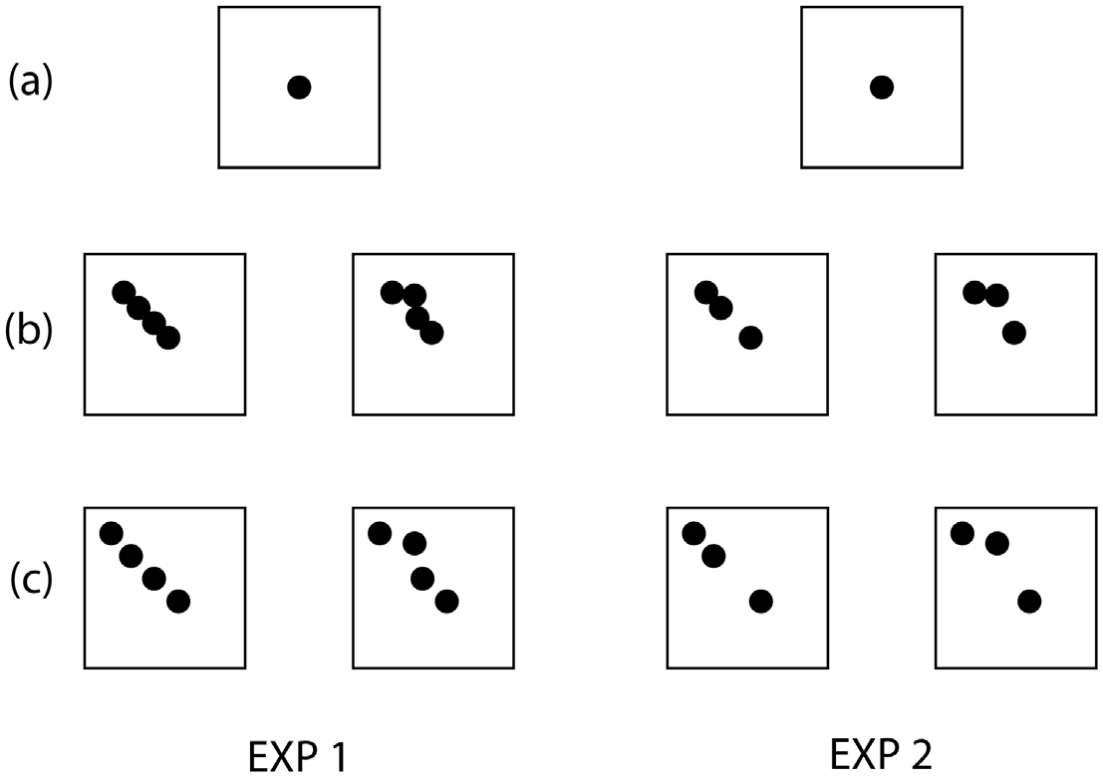
The experimental stimuli. The experimental stimuli used for the shaping (a), discrimination training (b) and the generalization testing phases (c) for Experiments 1 and 2. The main difference between aligned (BAL) and misaligned (IMBAL) conditions was the position of the second dot from the top, which was presented off-axis and therefor misaligned in the IMBAL condition. During the generalization phase, “spread apart” versions of the training stimuli, obtained by increasing the distance between the dots, were used (compare Figs. 2b and c., which allowed us to test whether BAL- and IMBAL-chicks differed in their generalization ability). In Experiment 2, fewer dots were presented thereby reducing the amount of information over the same spatial range and increasing axial noncoherence. If chicks demonstrated a consistent preference in both Experiments 1 and 2, this consistency could be attributable to an evaluation of implicit structure rather than a preference for the shape itself.

We trained chicks to peck for food reinforcement at one of two similar diagonal configurations of dots (inserted within a square frame). These differed only in the position of one of the dots (Fig. 2b), which was either aligned with the other dots on the diagonal axis of the frame or was misaligned with respect to the remaining dots and thus off-axis. Chicks reinforced on the former stimulus participated to the balanced (BAL) condition, whereas chicks reinforced on the latter stimulus participated to the imbalanced (IMBAL) condition. In a subsequent generalization phase, the chicks’ responses to modified versions of the stimuli (Fig. 2c) were tested to see whether there were any differences in performance between chicks trained on the aligned and misaligned stimuli. If this generalization was successfully achieved, and our expectations were confirmed, IMBAL chicks should show a clear category preference to peck the misaligned stimuli, while BAL chicks would show no clear preference. Two experiments were performed according to this procedure, the second of which reduced the number of display items maintaining overall stimulus size the same as the first experiment (Fig. 2 right panel). Reducing the amount of information over the same spatial range had the effect of increasing the amount of axial noncoherence and thus reducing specification of the relevant axis. If the chicks demonstrated a consistent preference in both Experiments 1 and 2, this consistency could be attributable to an evaluation of implicit structure rather than a preference for the shape itself.

## Results and Discussion

**Figure 3:**
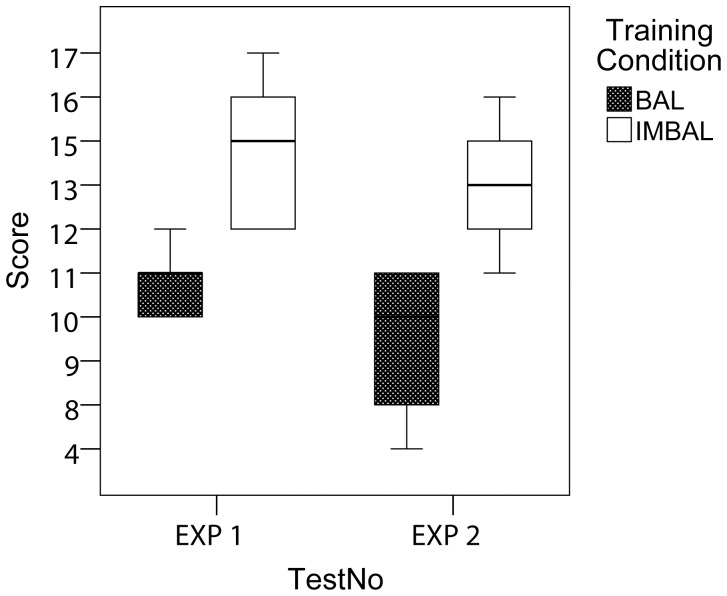
Results of Experiments 1 and 2. The proportion of trials (of 20) upon which chicks showed a preference to approach and peck the target stimulus in the generalization phase, as a function of training on aligned (BAL) or misaligned (IMBAL) training stimuli. The number of occasions upon which chicks showed a category preference consistent with their training was significantly different between the IMBAL- and BAL-chicks in both Experiments 1 and 2.

The number of occasions upon which chicks showed a category preference consistent with their training during the generalization phase was significantly different between the IMBAL- and BAL-chicks in Experiment 1 (t_10_ = −3.702, p<.005) and in Experiment 2 (t_6_ = −2.673, p<.05). However, Fig. 3 shows that in both experiments chicks’ performance was better than at chance (10 correct trials out of 20) in the IMBAL (Exp. 1, median = 14.17/20, t_5_ = 4.776, p<.005; Exp. 2, median = 13.5/20, t_3_ = 3.184, p = .05), but not in the BAL conditions (Exp. 1, median = 10.33/20, t_5_ = .598, p = .576; Exp. 2, median = 8.5/20, t_3_ = −.965, p = .406). Note that for Experiment 2, the data were log-transformed to normality to correct for asymmetric distribution.

Their performance indicates that preference for stimulus alignment generalizes beyond training only in the IMBAL chicks, while the “at chance” performance in the BAL chicks raises the possibility that a spontaneous preference for misalignment, elicited either by the training or by the testing stimuli, is a natural performance bias that reduces the effects of training using aligned stimuli. We considered the possibility that improved performance correlated with the number of trials needed to reach the learning criterion during the training phase and looked at the difference between these numbers in the BAL and IMBAL chicks. This difference was non-significant (Exp. 1, t_10_ = 1.38, p = .198; Exp. 2, t_6_ = −2.257, p = .101) indicating the chicks required a similar number of trials to achieve the learning criterion. We analyzed the groups for correct responses prior to criterion, and again found no significant differences (Exp. 1, t10 = 1.011, p = .336; Exp. 2, t6 = −1.101, p = .313). This result suggests that a difference in acquisition is not the key factor to explain the superior performance of IMBAL-chicks during the testing phase. However, on the grounds that not significant results should be interpreted with caution, we cannot definitively exclude an explanation in terms of a difference arising in the training phase, even if the existing evidence is against it. In any case, a difference in acquisition in favor of the off-axis group would be consistent with our overall interpretation of an advantage in the elaboration of the off axis display, and in particular with the presence of a spontaneous preference for misaligned configurations arising already in the training phase. In the present study our main aim was to study how structural balance/imbalance affected chicks’ performance during the generalization test. Future research might clarify whether any spontaneous preference for misaligned stimuli can be detected during training.

That the IMBAL chicks directed their pecking responses towards the misaligned stimulus significantly more often than chance demonstrates that chicks are able, in principle, to differentiate the stimuli according to their compositional structure. In these chicks, preference for misalignment is influenced but still significantly better than chance when the amount of axial noncoherence in the figure is increased. This indicates that preference for misalignment is only slightly affected by changes in the amount of information in the stimulus configuration. However, given that only the IMBAL chicks demonstrated this category preference, we propose that chicks tend to prefer misalignment, and treat the misaligned stimuli as if they are of more prospective interest than aligned stimuli. This argument is supported by a pilot study in which, during the generalization phase (but not during the training phase) frames were rotated by 45° while the figures were maintained with the alignment and axial coherence employed in Experiment 1. Given this rotation, all figures became misaligned relative to the structural skeleton of the frame. Data from 3 IMBAL-chicks (2 scoring 11 correct of 20 generalization trials, with the 3^rd^ scoring 10/20) and 2 BAL-chicks (both scoring 10/20 correct responses) revealed performance to be quite consistently at chance levels, suggesting that it is the relationship between figure and frame and not the mere presence of a frame that is required to induce the effects revealed in Experiments 1 and 2.

The possibility that the higher generalization performance of IMBAL chicks is actually due to the fact that the misaligned stimulus is perceived as more mealworm-like (edible) than the aligned one can be ruled out on various grounds: for instance as training stimuli, they both present a mealworm-like silhouette and if anything, the misaligned stimulus might appear more “unnatural” due to the pronounced curvature created by the displaced dot (incompatible with the physiology of most mealworms). As test stimuli, neither configuration can be claimed to resemble a mealworm due to the spacing introduced between the dots - particularly so for the 3-dot stimuli used in Experiment 2. In this experiment we should not have observed any advantage for IMBAL chicks if the mealworm-like appearance of the stimuli were the crucial factor determining the effect. Also, it should be noted that chicks used in this experiment were never fed with mealworms, nor had they the opportunity to have ever seen a mealworm (or other insect). Finally, in the pilot study using 45° rotated frames, we did not observe any preference for structural misalignment, as may have been the case if the chicks were influenced by the mere resemblance of one of the figures with any kind of mealworm.

The different performance of the IMBAL as compared to the BAL chicks can be explained both by a spontaneous preference for the misaligned stimulus interfering with testing performance of aligned chicks, as well as by a deeper encoding of stimulus properties, at training, in the misaligned group being expressed only later, during the test. Further research might help to decide between these two alternatives, but it should be noted that both of these explanations are consistent with our general hypothesis that chicks would show a higher interest for imbalanced relative to balanced configurations; interest in misalignment is not species-specific. Given major structural differences in the anatomy of the avian compared with the mammalian – and in particular the human - brain it is difficult to describe the similarity in preference in terms of activity within a particular brain region homologous between the two species (even though recent research reveals homologies between mammalian and avian brain structures, in particular with respect to the neocortex-like cognitive functions of the avian pallium [17]). Instead, these findings support the idea that the neurocognitive mechanism responsible for preferred structural imbalance is a major property of brain function, which we suggest relates to attentional deployment and the resulting dynamics of associated mechanisms rather than of the anatomical structure of the brain.

Consistent with other studies [9], our results suggest newly hatched chicks possess a sense of balance/imbalance and this relates to their responses to aligned and misaligned stimuli. If we are correct in our suggestion, chicks behave towards misalignment in a very similar way to humans. With this in mind, it is plausible to think that the mechanism responsible for engagement with or a preference for imbalance has evolved across changes in brain structure to better prepare the organism for the consequences of objects in the course of an unpredictable motion sequence. We hypothesise that possible transition states are also afforded by static objects that include cues to temporality, even implicit cues such as imbalance. In fact, the strength of these cues is such that information about the object is treated as secondary to information about its transitions [18]. A similar generalization is demonstrated by the IMBAL-chicks who show a preference for misaligned stimuli in spite of changes in their composition.

We have suggested the fundamental mechanism concerned with preference for structural misalignment is related to attention, however it may equally refer to memory or other higher order cognitive mechanisms without compromising comparison of the chicks’ with human behavior. This leads to a more radical hypothesis linking the preferential behavior of the chicks with preferential behavior in humans and in particular the “aesthetic response” to structural misalignment described by Arnheim [1] (illustrated by the displays in Figs. 1c, 1d and employed in the present study). As previously discussed, human interpretation of a composition can depend critically upon the positioning of elements to the extent that the positioning of a single item relative to its frame may be sufficient to influence judgments of “aesthetic quality” [1], [19]. It has been shown that aesthetic judgment may be resistant to alteration of the structural skeleton [20], but there is still supporting experimental evidence that regular, balanced compositions of the sort illustrated in Figs. 1a and 1b may be rated “aesthetic” as a consequence of the overall organizational simplicity afforded by the positioning of disk relative to frame [20], [21]. A higher aesthetic rating of simple forms is consistent with the primacy of organizational principles such as “simplicity” as well as the Gestalt concept of Prägnanz in which percepts will always be as ‘good’ as prevailing stimulus conditions allow [22]. Likewise and related to Arnheim’s claim there are experimental data that show static images which exhibit an implicit directionality (i.e. those including misalignment) are considered to be “better” when they afford motion towards a point of balance, particularly towards the center of a visual composition (illustrated with reference to [23] in Fig. 1b).

By our account and in the context of the simple preference behavior described in this study, there is no reason to believe that the human aesthetic response to structural misalignment is not based upon fundamentally similar mechanisms to those responsible for the preference behavior shown by chicks. This suggestion requires consideration in the light of contemporary theories of aesthetic preference and of animal preference. In the animal literature there are examples of preferential behavior resulting from reduced structural regularity: for instance, a higher degree of asymmetrical patterning (i.e. ‘fluctuating asymmetries’ in coloration or markings that indicate nutritional or energetic stress) can be an indicator of social dominance amongst European starlings (*Sturnus vulgaris*), particularly when competing for scarce food resources. This suggests that birds employ and understand the asymmetrical patterns as perceptual markers of their physiological status [24]. This could relate to the preference ratings of humans who preferred photographs of faces exhibiting a natural lateral (or directional) asymmetry relative to those in which this same asymmetry is digitally reduced [25]. However, not all evidence is consistent with the claim that preference for a conspecific (based upon aesthetic appeal) derives from a representation of asymmetry that is related to the fitness of the individual concerned: for example, the preference behavior of female zebra finches during mate selection favors males with more symmetrical plumage [26], [27] (and see [28] for a general review of preference behavior in birds). As reviewed above, the aesthetic response in human observers may favor either structural alignment or misalignment depending upon the composition concerned. This suggests aesthetic preference for compositional structure could be based upon at least two different, indeed fundamentally incompatible principles, perhaps employing different mechanisms and serving different functions. As exemplified in the literature reviewed above, in birds, symmetry is associated with positive trait selection while asymmetry is represented in terms of negative states (e.g. low energy or nutritional states). This tends to suggest that, in most cases, any preference for asymmetry could be a preference for the less fit individual or object. On this basis it could be argued that chicks prefer to peck at the imbalanced patterns because these best represent a physiologically compromised prey and so one which requires less effort to catch. Another function refers to our original idea that imbalance affords the potential for motion. In this case a preference for asymmetry offers the benefit that prey initiating an attempt to escape may be prioritized for response. Since the chicks’ prioritized response to misaligned stimuli concerns the positioning of the figure relative to its frame, we believe that the most likely explanation for the preference for imbalance reported here is related to implied motion rather than a general preference for asymmetry per se.

In either scenario, because the chicks employed in the present study had no prior experience of prey like patterns, preferential behavior towards misalignment must be based upon mechanisms that have evolved to automatically or reflexively bias behavior towards misaligned, asymmetrical or otherwise irregular forms. Related to aesthetics, this account is consistent with a psychological phenomenon referred to as the ‘peak shift effect’ [29]. Peak shift describes a preference for an exaggerated or “super normal” version of a more familiar object or stimulus and the tendency for chicks to direct attention and peck preferentially at the misaligned stimuli may represent a peak shift effect. If this is so, according to [29] chicks would employ a similar brain mechanism to that employed by the artist: as illustrated by Arnheim’s examples [1] an artist may choose to amplify or alter the physical dimensions of an item in an artistic scene in order to highlight essential features and thus convey a particular meaning. It has been suggested that peak shift, and by extension the aesthetic response to unusual visual form is one result of increased activation in extrastriate visual cortices in the artists and viewers’ brains, relative to activation induced by conventional or usual depictions [29]. A similar mechanism may explain preferences for novelty and complexity in art [30]. The aesthetic response is often reflexive and tends to require later introspection for proper realization or at least articulation. This is consistent with idea of a contribution of early sensory mechanisms because the output of those mechanisms, and therefore the basis for the aesthetic response, may not be directly accessible for conscious report [31]. Given the wealth of evidence that birds and humans process visual structure in a very similar way [8]–[11], [13], [16] both chicks and human observers could be argued to possess sensory mechanisms with sufficient capability to make available an aesthetic response to the stimuli presented in this study. However, because that response is commonly identified in terms of later introspection and is then expressed using modes such as language and defined according to emotional, social and cultural factors as well as physical characteristics of the item or composition, perhaps it is only humans who are able to conclusively demonstrate that they have experienced the aesthetic.

In addition and as already noted above, a clear parallel between chick and human behavior at the level of brain anatomy may be precluded by the fundamental differences in brain architecture between species. In this respect, it is perhaps at the level of neural dynamics that a more complete explanation for the relationship between peak shift and aesthetic preference will be found. Related to behavior, aesthetic pleasure may be considered a consequence of exploration and creative interpretation, the foundations of which relate to our tendency to seek solutions and resolve ambiguities in the environment. Whether chicks explore and resolve ambiguity in the misaligned stimuli as pragmatists or derive pleasure from their exploration remains unknown. If they found engagement with imbalance pleasurable, it could be argued that they experience the same sense of the aesthetic as does the human observer engaged by more complex artistic composition.

## Methods

### Ethics Statement

The experiments reported here comply with the current Italian and European Community laws for the ethical treatment of animals and the experimental procedures were conducted under ethics approval at the University of Padua licensed by the Ministero della Salute, Dipartimento Alimenti, Nutrizione e Sanità Pubblica Veterinaria (permit number 6723). Chicks were reared socially for the first five days and then individually from day 6. Food and water were available *ad libitum* in the rearing cages (moreover, during shaping and training chicks obtained food also in the experimental apparatus, as a reinforcement according to the experimental procedure).

### Participants

Participants were male domestic chicks (*Gallus gallus domesticus*) of the Hybro strain (N = 12 and 8, in Experiments 1 and 2 respectively) hatched from fertilized eggs obtained weekly from a local commercial hatchery (Agricola Berica, Montegalda (VI), Italy). Chicks were food deprived during the 2 h immediately before a training or a testing session (water deprivation was never administered).

### Apparatus and Stimuli

All stimuli were printed on a rectangular white paper base (9×6 cm). Each stimulus consisted in a configuration of grey dots (Ø 4 mm) within a square black frame (3.2×3.2 cm). During the initial shaping phase a stimulus representing a single central dot was used (Fig. 2a). During the discrimination training phase stimuli consisted in one aligned and one misaligned (one item offset from axis by ½ of its radius) configuration of 3 or 4 adjacent dots (in Exp. 1 and 2 respectively, Fig. 2b). In order to eliminate the possibility of cross-axial alignment, the item closest to the frame was distanced 1/8 of the frame's length (and height) from the bounding line. The upper left quadrant was selected due to its potential for enhanced relevance and augmentation of the effect of structural misalignment, because this location optimally exploits the effects of visual gravity [1], [21]. The main difference occurring between the two training stimuli was the position of the second dot from the top, which in the misaligned configuration was off-axis (see Fig. 2b). Both configurations of dots used as training stimuli might appear as edible objects to chicks, since their silhouette resembles that of a mealworm or insect grub. Finally, during the generalization phase, “spread apart” versions of the training stimuli, obtained by increasing the distance between the dots, were used (compare Figs. 2b and c, which allowed us to test whether BAL- and IMBAL-chicks differed in their generalization ability).

**Figure 4:**
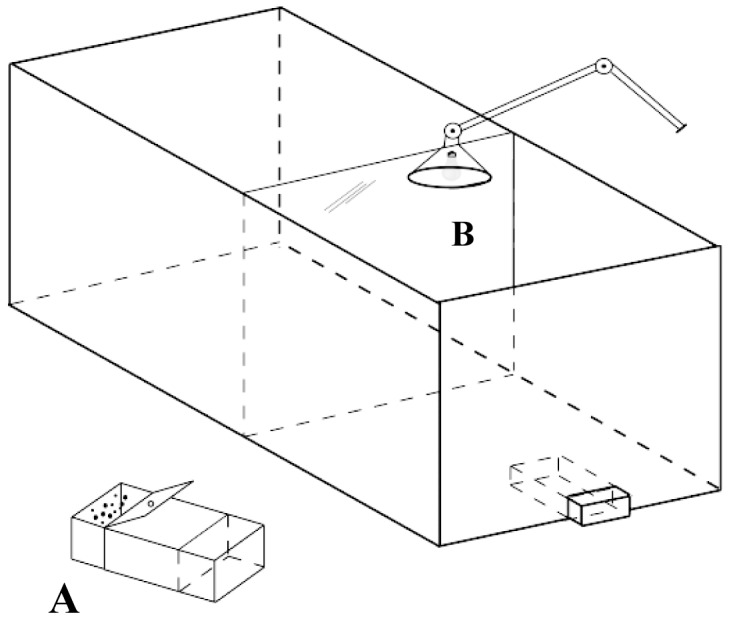
A schematic representation of the apparatus. The food-box is denoted by A while B indicates the movable partition. A movable lamp providing illumination to the inside of the apparatus is represented above it. At the beginning of each trial, removing the partition, we allowed the chick to reach for the food box. This figure represents the initial training, when chicks are shaped to peck to the one-dot stimulus (Fig. 2a): the chick is rewarded (by opening the food box for a few seconds, allowing the ingestion of some food grains) for each peck at the stimulus. Then the experimenters close the food box and confine the chick behind partition B (the starting position for the following trial). In the subsequent discrimination training two food boxes are presented side by side, and the chick has to choose which one to peck on the basis of the stimulus displayed on it (an aligned or a misaligned configuration of dots, Fig. 2b). After a correct response (a peck on S+) the animal is rewarded (see above), whereas after an incorrect response the chick will be confined behind partition B for 15 s without access to food. The procedure used at test differs only in that the chick is always reinforced for each peck at either stimulus (see Fig. 2c).

The experimental apparatus (Fig. 4) consisted of a rectangular white-painted cage (33×38×60 cm) with a slit at the bottom of one of the short walls through which the food-box (6×6×12 cm) could be introduced. The food-box had a drawer that could be pushed open from outside of the cage by the experimenters in order to allow access to the food. The stimuli were fixed on the top of the food-box (at 45°).

### Design and Procedure

Shaping started in the morning of day 8. Each chick was trained to peck at a stimulus, which was placed over the food box, in order to open the food-box drawer. In this phase the single dot stimulus was used (Fig. 2a). Shaping was considered complete after the chick had promptly pecked at the dot during 10 consecutive trials. From day 8 to approximately day 11 or 12, chicks underwent discrimination training. During this phase the chicks were presented with two identical boxes, each one associated with a different stimulus (one misaligned and one aligned configuration formed of 4 adjacent dots in Exp. 1 and of 3 dots in Exp. 2, see appropriately labeled vertical panels in Fig. 2). Half of the chicks were assigned to the aligned condition (BAL) and half to the misaligned condition (IMBAL). Chicks of both groups underwent to a discrimination training: they had to learn to peck at one stimulus (S+), avoiding the other one (S-). For IMBAL-chicks, the misaligned configuration was the positive stimulus (S+): only pecks to that configuration were reinforced by opening the food box (when the chick pecked at the other stimulus, S- it was gently pushed back with a mobile partition and, after 15 s, it was released for the next trial). The opposite was true for BAL-chicks, for which the aligned configuration was the S+ (reinforced stimulus) and the imbalanced configuration was the S-.

During the training phase, the food reinforcement was delivered by opening the food drawer for a few seconds (the time needed by the chicks to ingest approximately a few grains of food, flexibly adjusted according to each subject’s behavior). Reinforcement was terminated by gently closing the food drawer and by then confining the subject in the back of the apparatus, thanks to the movable partition that was used to carefully “push” the chick (Fig. 4 B). Trial duration was flexibly determined according to the subjects’ behavior (the time required by each subject to peck one of the two stimuli). Similarly, the inter-trial interval varied according to the time needed by the experimenters to swap stimuli’s positions.

During the learning phase, the left-right position of the two stimuli changed from trial to trial according to a semi random sequence (LLRRLRLRLLRRLRLRLLRR). This is a standard sequence, initially developed by [32] and subsequently employed in a number of other studies with domestic chicks [33]–[35]. To begin and in order to make it easier for the chicks of both groups to learn the discrimination successfully, the positive stimulus was rendered more perceptually distinctive. In order to do so, one of the grey dots in the positive stimulus (the second dot from above, whose position differed between the aligned and the misaligned configuration) was substituted by an identical sized red dot. An advantage of this procedure is that the position of the crucial “target” dot, i.e. the distinctive feature that differentiated the positive stimulus from the negative one (regardless of whether this was the balanced or the imbalanced configuration), was rendered perceptually salient for chicks of both groups starting from the very beginning of the discrimination training. When the chick had achieved this facilitated discrimination according to a flexibly defined learning criterion, it was retrained with an identical pair of stimuli both composed of grey dots only. This discrimination-learning phase stopped when the chick had pecked at the correct stimulus in 17 of 20 consecutive trials (i.e. the learning criterion was reached). About 1 h after the end of the training phase, chicks underwent a generalization-phase consisting in 20 consecutive trials during which pecks at either stimulus were reinforced (the left-right position of the stimuli was changed from trial to trial according to a semi random sequence, see above). The number of correct choices made by each animal was recorded. Even though at test all choices were rewarded, for data coding we defined as a correct choice a peck to the spread-apart version of the configuration that was reinforced at training. The experimenter running the task and doing the scoring was unaware of the identity of the chick being tested. Moreover, the pecking response used as dependent variable is a very pronounced and discrete behavior, unlikely to be liable to influences or misinterpretations. Also, testing modalities involved no direct manual interaction between the animal and the experimenter (interaction was always indirect, thanks to the use of the movable partition).
